# Multirotor Motor Failure Detection with Piezo Sensor

**DOI:** 10.3390/s23021048

**Published:** 2023-01-16

**Authors:** Leszek Ambroziak, Daniel Ołdziej, Andrzej Koszewnik

**Affiliations:** Department of Robotics and Mechatronics, Faculty of Mechanical Engineering, Bialystok University of Technology, Wiejska St. 45C, 15-351 Bialystok, Poland

**Keywords:** motor failure, detection, diagnostics, piezo sensor, vibration analysis, unmanned aerial vehicles, multirotor

## Abstract

Failure detection of Unmanned Aerial Vehicle (UAV) motors and propulsion systems is the most important step in the implementation of active fault-tolerant control systems. This will increase the reliability of unmanned systems and increase the level of safety, especially in civil and commercial applications. The following paper presents a method of motor failure detection in the multirotor UAV using piezo bars. The results of a real flight, in which the failure of the propulsion system caused the crash of a hybrid VTOL UAV, were presented and analyzed. The conclusions drawn from this flight led to the development of a lightweight, simple and reliable sensor that can detect a failure of the UAV propulsion system. The article presents the outcomes of laboratory tests concerning measurements made with a piezo sensor. An extensive analysis of the obtained results of vibrations recorded on a flying platform arm with a propulsion system is presented, and a methodology for using this type of data to detect failures is proposed. The article presents the possibility of using a piezoelectric sensor to record vibrations on the basis of which it is possible to detect a failure of the UAV propulsion system.

## 1. Introduction

Over the past decade, Unmanned Aerial Systems (UAS) have advanced greatly, mostly due to the dynamic progress in robotics, control engineering, telecommunication, and material sciences [1,2,3]. Thus, it significantly expanded the field of application of unmanned systems, especially the civil one [4,5,6,7]. UAS are considered a complete system usually composed of the following components: Unmanned Aerial Vehicle (UAV), ground control station (GCS), command and control data link (C2 Link), human ground UAV operator (UAVO), and the payload/instrumentation, such as optical head, various kinds of sensors, etc. Sometimes the UAS is equipped additionally with very specialized components such as a launch and recovery system, ground technical supply vehicle, docking station, power generators, antenna trackers, or radars. In the mentioned statement, the UAV is a critical component, and its ability to confidently and safely perform airspace missions is necessary. One of the most important issues that has been developed and studied intensively is fault tolerant control systems (FTCS) dedicated to different types of unmanned aircraft [8,9,10,11], which must be accurately mathematically modeled before implementation [12,13]. FTCM can be divided into active and passive kinds. The passive ones mainly concern the fail-safe functions implemented in the event of various errors, such as no/weak GPS signal [14,15] or no communication with the ground station [16]. In this case, the UAV simply returns to the launch point (home position), goes into hover mode, or simply loiters/circles around a specific point (last known position). Active failure tolerant systems reconfigure the control system, and change the control algorithm or control loop parameters [17,18]. Examples of such systems are the propulsion system failure response systems used in multicopters (MC). This is due to the fact that drive failure is the most common cause of UAV crashes, which can be predicted, detected, and even prevented. Very often, in such cases, for example, the control system switches from a quadrocopter to a tricopter configuration. The most important element of such a system is the moment of detecting failure.

In this article, we have attempted to determine the possibility of using a piezoelectric sensor to detect engine failure. An analytical description of vibrations recorded using a piezoelectric sensor placed on the UAV arm is presented in this paper. The presented results of experimental research showed a strong correlation between the moment of motor failure injection in a significant increase in the registered vibrations level. The work also verified the influence of the location of the sensor on the recorded vibrations.

The remainder of the paper is organized in the following manner: Section 2 includes the description of our motivation for research on a lightweight system for early detection of UAV drive failures as a necessary element to improve the safety and quality of operation of this type of aircraft. Section 3 describes the UAV we have worked on previously that had an in-flight failure that could have been avoided if it had been equipped with an appropriate drive abnormality detection system. Section 4 contains the results of VTOL flight tests that were carried out over the water reservoir where engine failure occurred. The idea of the drive damage detection system using the piezoelectric sensors proposed by us is described in Section 5. Experiments of the motor in a laboratory concerned various throttle levels with simulated cyclical occurrence of damage, and its detection using a piezoelectric element is presented in Section 6. Section 7 contains a summary of our work and the legitimacy of the solution adopted for unmanned aerial vehicles with the risk of propulsion failure.

## 2. Research Motivation

UAVs in multicopter (MC) configuration (quadrotor, hexa rotor, hybrid UAVs called quad-planes or separate lift and thrust (SLT) VTOL (vertical take-off and landing) are widely used types of frames in many civil, and military applications [19]. For energy-saving efficiency, quadrotors are most suitable for various applications. Additional 4 motors can easily provide vertical take-off and landing functions to quadplanes, ensuring the simplicity of a VTOL design solution. Quadrotors are now widely available, both as commercial products and as open-source projects. However, one of their significant disadvantages is the lack of resilience to rotor failures (electric motor or propeller damage, ESC, power supply errors). This is because quadrotors are underactuated systems, and the loss of one or more actuators results in a loss of controllability and eventual destabilization, when operating with a standard control system.

**Figure 1:**
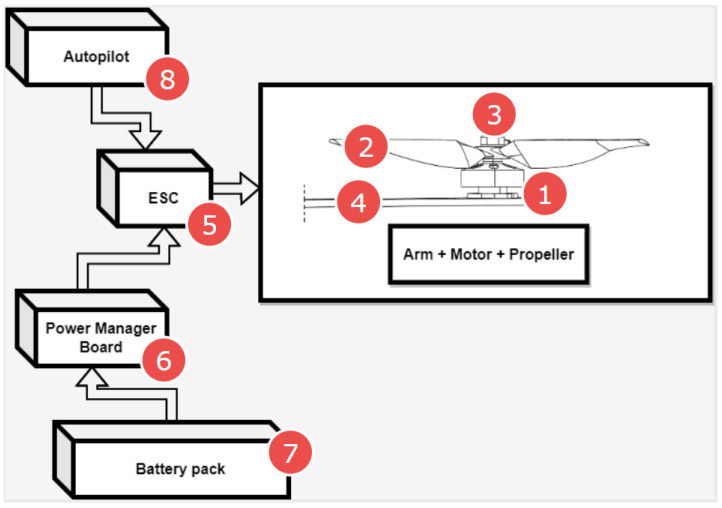
Multicopter propulsion system scheme and potential failure sources (1—Electric motor, 2—Propeller, 3—Propeller hub and nut, 4—Motor arm, 5—Electronic Speed Controller (ESC), 6—Power manager Board, 7—Battery pack, 8—Autopilot and flight control system).

Figure 1 shows a diagram of the multicopter propulsion system. It also can be a scheme where there may be damage caused by dangerous falls and destruction of this type of flying object. The most common problems concern the motor itself (mostly bearings, winding), the propeller, propeller mounting (nut) or the multicopter arm where the engine-propeller pair is fixed (Figure 1, No. 1–4). The potential problems with propulsion system can also be caused by battery pack-sudden voltage drop, overheating of wires (Figure 1, No. 7), power manager board-overheating of PCB, overheating of soldered joints (Figure 1, No. 6). A large number of potential error sources in the propulsion system of a multicopter are associated with the ESC (Figure 1, No. 5). Sometimes errors relate to control signals produced by an autopilot (Figure 1, No. 8). To increase the level of reliability and safety of quadrotors, many methods have been employed, which are related to control system reconfiguration or using fail-safe mechanisms like parachutes or airbags. However, the use of all these systems is impossible without a precise and fast determination of the time when failure occurs. To detect the precise time of failure, many methods can be applied. One of the simplest methods of detecting motor failure is to use an electronic speed controller (ESC) of the motor with RPM measurement [20]. Such ESCs are usually larger and heavier than normal. Unfortunately, this method is not free from disadvantages. In that way, it is difficult to detect all types of failures that may occur during the UAV flight. In this approach, the drop in rotational speed can be easily monitored. Most often, this drop in rotational speed is related to, for example, the total stopping of the motor in the event of a power supply failure. Other cases like partial damage of the propeller, bearing problems, and loss of one or two power phases in the ESC cannot be accurately and precisely detected with this method. In this case, the approach described in [21] may seem interesting, where the fault characteristics and robust observer were applied to the detection and isolation of fault source in the electric motor. A significant part of UAV propulsion system failures concerns precisely such situations where we do not have a complete loss of rotational speed. The authors of this article dealt with many similar situations during the implementation of the last research and the development of the project, which concerned the use of UAVs of the VTOL type in collision-free navigation of sea-going vessels [22,23]. In this project, one of the aircraft used was the hybrid VTOL (quadwing airframe). The relatively large weight of the object, large disturbances from winds reinforced by the wing surface, and sea operating conditions very often caused problems like some damage to the vertical take-off and landing power unit (4 rotors for multicopter operations). One such flight with a crash was video recorded and presented here https://youtu.be/QXs3K2mL8Zs (accessed on 4 December 2022). All these reasons were the motivation of our research to look for a method with which anomalies in the multirotor motor behavior could be detected early enough. Early failure detection would allow for:

- the damage location,
- classification of the level of damage and possible danger (with additional analysis of flight parameters),
- taking an appropriate response, such as continuing the flight, emergency landing, or reconfiguring the steering control algorithm to resolve the fault,

The above also means:

- increased safety of usage,
- minimization of repair costs resulting from damage or breakdown of the UAV,
- expanding the possibilities of using UAVs in new fields of life.

## 3. Hybrid VTOL UAV

**Figure 2:**
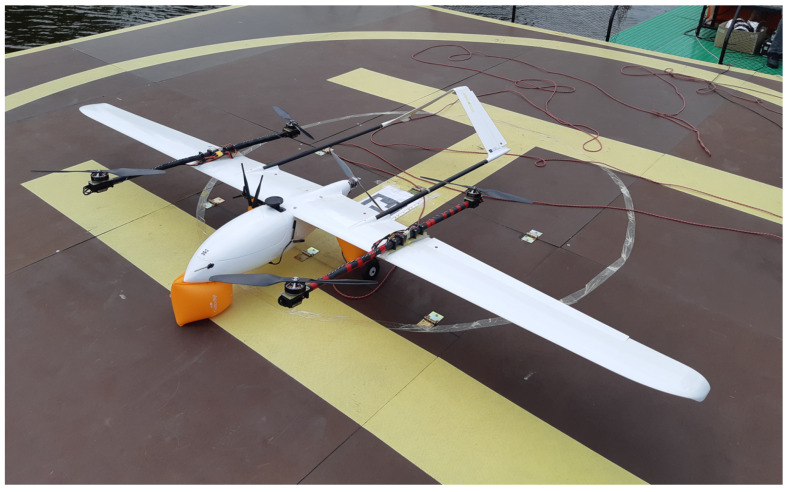
The Albatross VTOL UAV.

The subject of research is a hybrid unmanned aerial vehicle called Albatross (Figure 2) and its propulsion system. Albatross is a commercial fixed-wing platform customized to vertical take-off and landing by adding special wing brackets and tail booms with four motor mounts. The airframe is equipped with an inverted V-tail and the main dimensions are as follows: width—3 m, length—1.7 m, maximum take-off weight (MTOW) 8 kg. Albatross is made of carbon fiber and fiberglass composite.

The Albatross was developed as a part of a maritime project related to maritime safety [22]. That is why it was equipped with inflatable airbags to prevent drowning in the event of an unexpected landing on the water. The Albatross propulsion system is based on 4 electric motors for VTOL functions and 1 electric pusher-type motor for fixed wing (FW) operations. It gives classical quadwing VTOL construction. Important features of the Albatross are the on-board equipment which provides:

- autopilot allowing automatic flight control and the implementation of the function of switching from/to airplane mode to/from multicopter mode, following the mobile landing platform (ferry, ship, mobile robot), precise automatic landing on the mobile landing platform,
- on-board computer communicating with the autopilot by cable, responsible for the tracking and estimation of the position of the mobile landing platform and precise positioning of the flying object,
- vision computer responsible for image processing related to precise landing and airstrip monitoring to perform the collision avoidance function.

**Figure 3:**
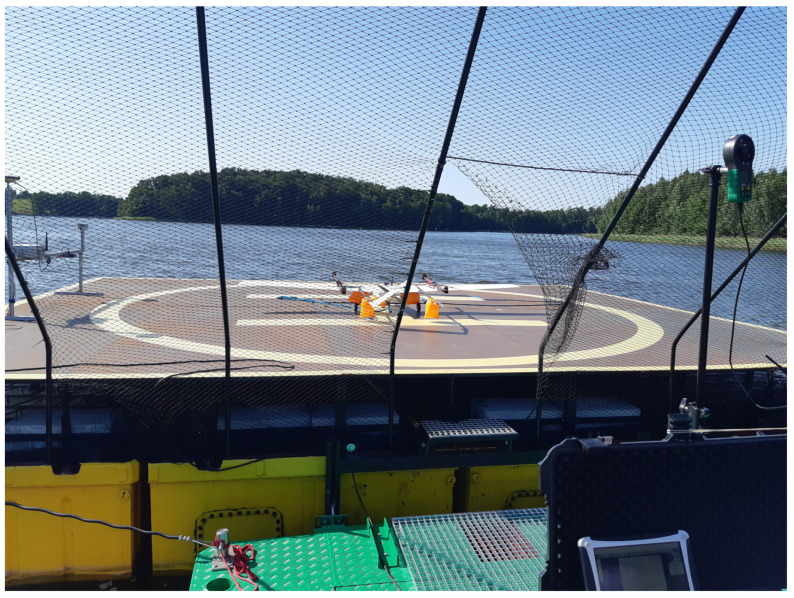
Albatross VTOL UAV during tests in quasi-real conditions on a lake.

The Albatross maximum speed is 30 m/s—in fixed-wing mode. The flight range is about 100 km (depending on the capacity of the battery pack, take-off conditions, and mode of the take-off phase and flight conditions). Maximum flight time is up to 1 h (depending on weight, flight scenario, take-off conditions and mode, and atmospheric conditions). The Albatross platform was prepared as a test platform in a project concerning cooperation between an autonomous sea vessel and a UAV. In this project, the VTOL platform was a sensor carrier providing image data about the sea situation to an unmanned sea vessel. The Albatross prototype platform was not the final version of the VTOL in this mentioned R&D works, but only a 1:1 scale model that allowed the reproduction of the dynamics of such an object and testing all functions of control and communication system in quasi-real (on the lake) and real conditions (at sea). Figure 3 shows the Albatross during tests on the lake. They took place on a specially prepared floating landing pad. Whole VTOL operations and flight tests took place from this landing pad. The Albatross platform is a relatively large platform due to its weight and wingspan. The power supply and propulsion system were completely redesigned relative to the original Albatross airframe. During the vast number of tests, there were several failures of the propulsion system, which was the main motivation for this article. Importantly, in other types of flying platforms (e.g., hexarotors with a mass of around 10 kg) used in our project, such events were also noticed.

## 4. Hybrid VTOL Flight with Propulsion System Failure

**Figure 4:**
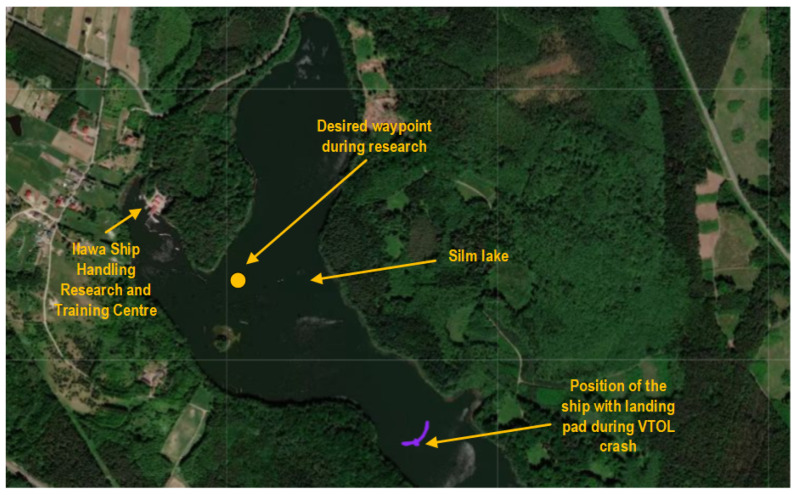
VTOL UAV research area during quasi-real tests.

The main motivation for undertaking this research on the early detection of multicopter propulsion system failures was the problem with ESCs and motors occurring during a large number of VTOL tests. This section will present the results of such a failed flight (this is not a common practice in scientific papers), which was caused by an ESC failure. These tests were carried out over the water reservoir—Lake Silm near Iława city in Ship Handling Research and Training Centre (Figure 4). The Albatross aircraft was not equipped with any detection system for incorrect operation of the multirotor propulsion system (rpm sensor, thrust and torque sensor, or any other type of detecting method). The ill-fated flight involved performing the following stages of the mission:

- automatic take off from mobile landing pad (while moving) in MC mode,
- following mobile landing pad with automatic follow-me function in MC mode,
- transition from MC mode to FW mode,
- navigation to waypoint located far from mobile landing pad in FW mode,
- return in FW mode to the landing pad
- transition from FW mode to MC mode
- transition from MC mode to FW mode,
- following mobile landing pad in MC mode,
- automatic landing in MC mode on a landing pad (while moving).

**Figure 5:**
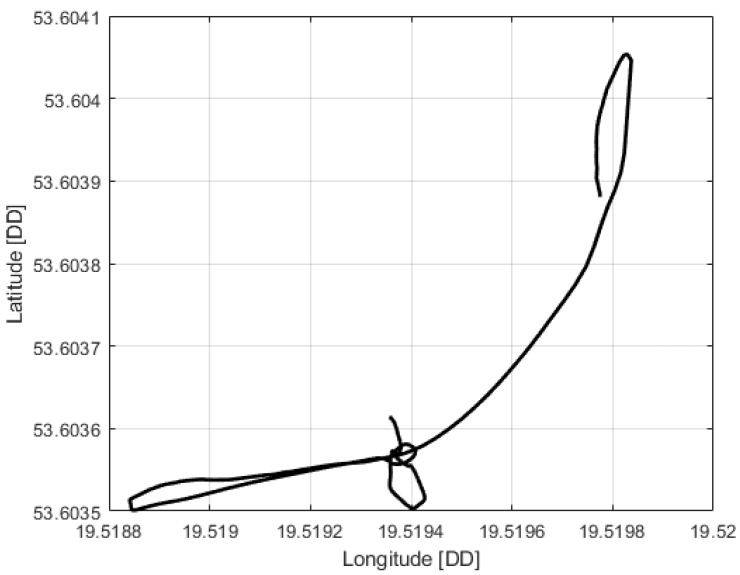
VTOL UAV trajectory during crash flight.

**Figure 6:**
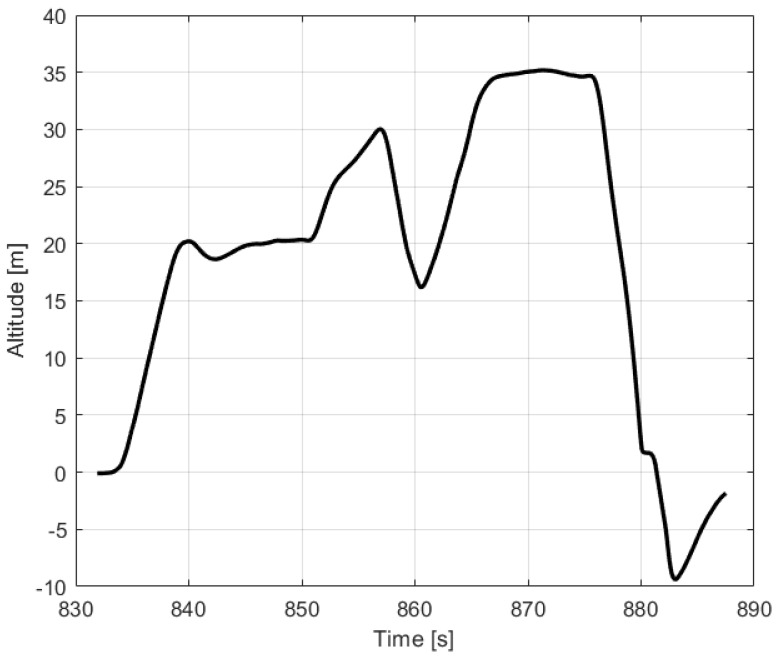
Altitude profile of VTOL UAV during crash flight.

**Figure 7:**
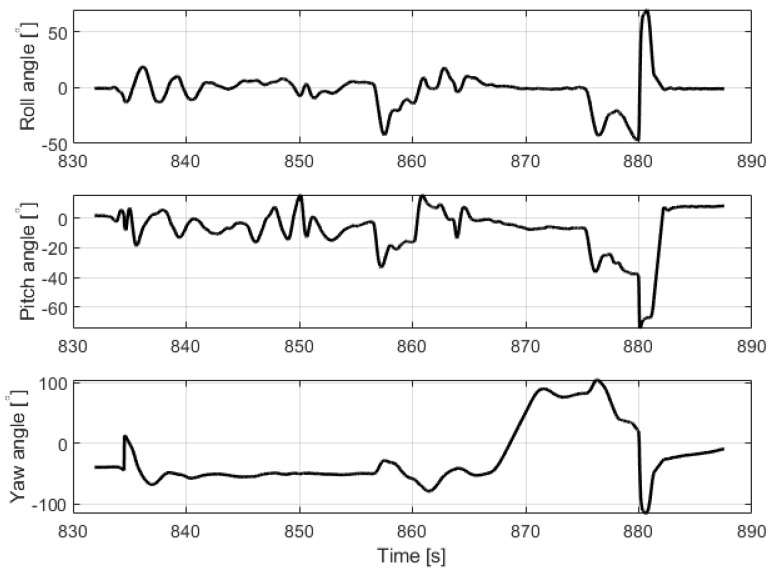
Roll, Pitch and Yaw angles of VTOL UAV during crash flight.

**Figure 8:**
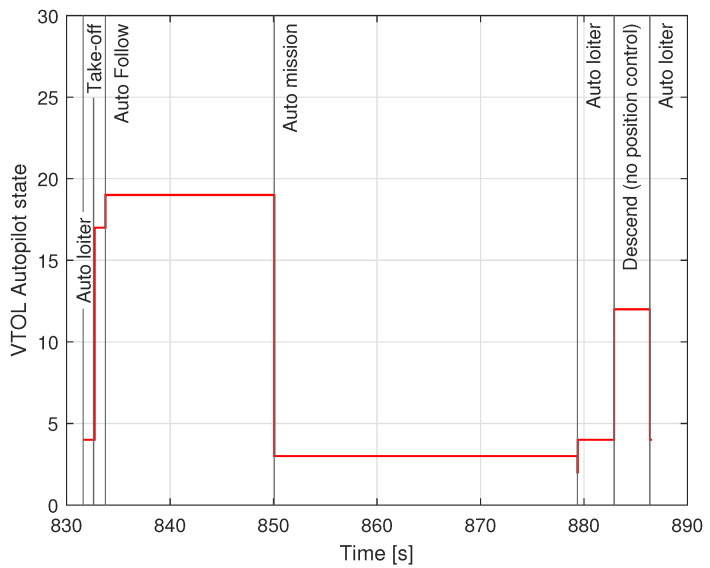
VTOL UAV states during crash flight.

The first stage-automatic take-off was successful. The plane climbed to a given altitude of 20 m. Next, the VTOL was switched to the mobile platform following the function, but this task was not completed. In this phase, problems with proper tracking of the desired position were noticed. Uncontrollably, VTOL moved to the left side of the mobile platform, then to the right side. Soon after, the VTOL UAV began to lose altitude and fell into the water. The trajectory recorded during this flight is shown in Figure 5. Analyzing the presented trajectory, one can easily relate it to the film presented by the accident. VTOL, instead of flying to the desired waypoint with coordinates 53.607 and 19.511 at an altitude of 35 m, was moving around the landing platform in an uncontrolled manner. Figure 6 presents the flight altitude profile. Only the first stage, in terms of reaching the desired altitude, has been completed. The VTOL reached the desired 20 m during the take-off, then tried to reach the desired 35 m of altitude; however, after a short ascending (to an altitude of around 30 m), it reduced the altitude to about 15 m and then reached the desired 35 m. XY position control was not maintained, and even the altitude was not followed precisely. There was also no automatic transition from the MC to FW mode. The climax came at 875 s into the mission when the VTOL suddenly began to lose altitude. The moment of impact occurred in the 880th second of the mission. This can be noted in the altitude (Figure 6) and Roll, Pitch, and Yaw angle values (Figure 7). From the time plots of the Pitch and Roll angles, it can be concluded that in the last phase of the flight, the VTOL pitched strongly and rolled to the left side. This could indicate a problem with the left front MC mode motor. In addition, Figure 8 shows the autopilot flight modes during the VTOL mission. The first phases of the mission—take-off, follow, and mission mode—were triggered automatically by the autopilot. Switching to loiter function was made by the safety RC pilot but without any manual control options. The autopilot analyzing the failure situation automatically switched VTOL to descent mode without position control.

## 5. The Idea of Motor Failure Piezo Sensor

The propulsion of the unmanned aerial vehicle considered in this article is based on a set of propellers, a BLDC electric motor, a BLDC motor controller (ESC), and an electric power source. The executive element, i.e., the motor and the propeller, generates vibrations during its operation (even normal). The frequency and amplitude of vibrations depend on the flight phase and weather conditions, but also on the internal condition of the drive and propeller. Frictional resistance from dirty or worn bearings or defects (damaged) propellers dramatically changes these vibration characteristics. A sudden failure caused by a propeller breaking off or the engine falling out can, therefore, be quickly noted if we use an appropriate detection system. Our idea is to use piezoelectric elements already successfully applied in energy harvesting (EH) applications [24,25], structure damage monitoring [26] and also implemented and tested on a UAV [27]. The piezoelectric sensor generates a variable voltage that can be related to the frequency and amplitude of the deflection. Therefore, in our opinion, it is possible to deduce the condition of the correct operation of the drive or the drive with impending or occurring damage. Thus, if necessary, appropriate actions can be taken, such as reconfiguration of the controls to ensure flight safety.

**Figure 9:**
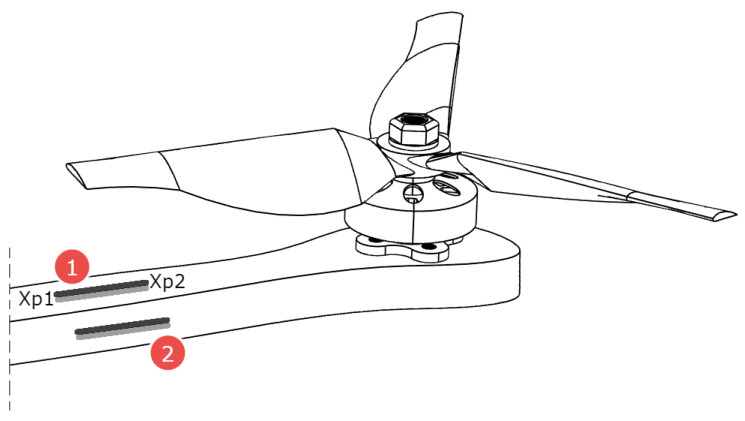
Mounting places of the piezo sensor (1—upper location, 2—side location).

Taking into account the proposed idea, it is known that a single arm of the UAV frame with an integrated piezo harvester is excited to vibration by lifting force $F_{\mathrm{lift}\_\mathrm{force}}\left(t\right)$ generated by the propulsion system locating on the free end of this arm (see Figure 9). Then, the general equation of transverse vibration of this mechanical structure with the piezo harvester is used to monitor the fault propulsion system at position (*x*${}_{P1}$,*x*${}_{P2}$, Figure 9) can be expressed in the following form [27]:

(1) $$\begin{array}{c} EI\frac{\partial^{4}w\left(x,t\right)}{\partial x^{4}}+m_{pipe}\frac{\partial^{2}w\left(x,t\right)}{dt^{2}}-\rho_{P}t_{P}\frac{\partial^{2}w\left(x,t\right)}{dt^{2}}\\ -\Gamma V\left(t\right)\left[H\left(x-x_{P2}\right)-H\left(x-x_{P1}\right)\right]=\\ F_{\mathrm{lift}\_\mathrm{force}}\delta \left(x-x_{0}\right) \end{array}$$

where: ρ${}_{P}$—mass density of the piezo harvester, *t*${}_{P}$—thickness of the piezo harvester, *δ(x)*—Dirac function along the *X* axis, *V(t)*—voltage flowing through the external resistive load *R*, Γ—electromechanical coupling factor (${\tilde{\Gamma }}_{n}=-E_{P}d_{31}w_{P}t_{c}$).

The mentioned piezo-harvester requires considering also the approach from the electrical point of view. To do this, the electrical charge accumulated at its electrodes can be calculated over the whole surface area in the following form:

(2) $$Q=\int_{P1}^{P2}\left(d_{31}E_{P}{\bar{\delta }}_{P}+\varepsilon_{33}E_{3}\right)w_{P}dx$$

where: ε${}_{33}$—permittivity at constant stress, *d*${}_{31}$—piezoelectric strain coefficient, *E*${}_{P}$—Young modulus of the piezo-electric material, ${\bar{\delta }}_{P}$—bending strain along the middle surface of the piezo layer, *w*${}_{P}$—the width of the piezo harvester, *E*${}_{3}$—electric field.

Next, applying Ohm’s law, the current flowing through the load resistor *R* can be expressed as:

(3) $$i\left(t\right)=\frac{V(t)}{R}=\frac{d}{dt}\left[\int_{P1}^{P2}\left(d_{31}E_{P}{\bar{\delta }}_{P}+\varepsilon_{33}E_{3}\right)w_{P}dx\right]$$

where: *V*(*t*)—AC voltage generated from the piezo-harvester, *R*—resistive load applied to the system.

Taking into account Equation (3), it can be seen that the current *i(t)* is strongly associated with strains of the piezoelectric harvester and the electrical field applied to its electrodes. This caused the electrical circuit of the proposed system to be expressed in the following form [24]:

(4) $$C_{p}\frac{dV(t)}{dt}+\frac{V_{p}}{R}+\Gamma \left[\int_{x_{P1}}^{x_{P2}}\frac{\partial^{3}w\left(x,t\right)}{\partial x^{2}\partial t}dx\right]=0$$

where: *C*${}_{p}$—capacitance of the piezo-patch harvester $C_{p}=\frac{\varepsilon_{33}w_{P}l_{P}}{t_{P}}$.

Both equations, Equations (1) and (4), refer to the lumped electro-elastic model parameters of the piezo-harvester integrated into a one-dimensional mechanical structure in physical coordinates. However, from the energy harvesting point of view, it should be analyzed in modal coordinates. To do this, displacement of the host structure is represented as the multiplication of an absolutely and uniformly convergent series of the eigenfunctions in the following form:

(5) $$w\left(x,t\right)=\sum_{n=1}^{\infty }\phi_{n}\left(x\right)\eta_{n}^{}\left(t\right)$$

where: $\phi_{n}\left(x\right)$—the mass normalized eigenfunction (mode shapes), *η${}_{n}$(t)*—the modal coordinate of the considered frame for *n*th mode.

The considered structure represents the cantilever beam. It caused that eigenvectors of this structure, after considering the boundary coordinates, given in Equation (6) split by geometric and time variables, what can be expressed as:

(6) $$\begin{array}{c} w\left(0,t\right)=0,{\left.\frac{\partial w\left(x,t\right)}{\partial x}\right|}_{x=0}=0,{\left.EI\frac{\partial^{2}w\left(x,t\right)}{\partial x^{2}}\right|}_{x=l}=0,{\left.EI\frac{\partial^{3}w\left(x,t\right)}{\partial x^{3}}\right|}_{x=l}=0 \end{array}$$

(7) $$\begin{array}{c} \phi_{n}\left(x\right)=\left(\mathrm{sh}\lambda_{n}L_{arm}+\sin\lambda_{n}L_{arm}\right)\left(\mathrm{ch}\lambda_{n}x-\cos\lambda_{n}x\right)-\\ \left(\mathrm{ch}\lambda_{n}L_{arm}+\cos\lambda_{n}L_{arm}\right)\left(\mathrm{sh}\lambda_{n}x-\sin\lambda_{n}x\right) \end{array}$$

where: $\lambda_{n}L_{arm}=\frac{2n-1}{2}\pi$ for *n* = 1, 2, 3.

The obtained Equation (7) put into Equation (1) leads to solving the eigenvalue problem of the smart beam for short circuit conditions. Then, the natural frequency ${}_{n}$ of the considered structure for *n*th mode can be presented in the following form [28]:

(8) $$\omega_{n}=\lambda_{n}^{2}\sqrt{\frac{E_{arm}I_{arm}}{m_{arm}L_{arm}{}^{4}}}$$

where: $\lambda_{n}^{}$—frequency parameter of an undamped structure, *E*${}_{arm}$—Young modulus of the UAV arm, *I*${}_{arm}$—inertia moment of the UAV arm, *m*—the mass of the arm, *L*${}_{arm}$—the length of the arm.

Taking into account the modal analysis procedure for this structure, an electromechanical coupled ordinary differential equation for the modal time response $\eta_{n}$ can be expressed as

(9) $$\frac{d^{2}\eta_{n}\left(t\right)}{dt^{2}}+2\xi_{n}\omega_{n}\frac{d\eta_{n}\left(t\right)}{dt}+\omega^{2}{}_{n}\eta_{n}\left(t\right)+{\tilde{\Gamma }}_{n}v\left(t\right)=f_{n}\left(t\right)\delta \left(x-x_{0}\right)$$

where: $\xi_{n}$—modal damping ratio, $f_{n}\left(t\right)$—modal force applied to the structure.

As a result, the modal electromechanical coupling term ${\tilde{\Gamma }}_{n}$ can be presented as:

(10) $${\tilde{\Gamma }}_{n}=-E_{P}d_{31}w_{P}t_{c}\int_{0}^{l}\frac{d^{2}\phi_{n}\left(x\right)}{dx^{2}}dx=-E_{P}d_{31}w_{P}t_{c}{\left.\frac{d\phi_{n}\left(x\right)}{dx}\right|}_{x=l}$$

The obtained modal electromechanical coupling term given by Equation (10) and the vertical deflection from Equation (6) put into Equation (4) lead to modifying the electrical circuit equation in the following form:

(11) $$C_{p}\frac{dV(t)}{dt}+\frac{V}{R}-\sum_{n=1}^{\infty }{\tilde{\Gamma }}_{n}\frac{d\eta_{n}\left(t\right)}{dt}=0$$

The performed considerations of the considered structure with an integrated piezo-harvester for modal coordinates allow voltage calculation generated by the piezo for vibrating structure as well as monitoring the propulsion system to fast detect the fault.

Using the piezo sensor, we can record changes in vibrations characteristics in the drive system due to:

- propeller unbalance (diameter change caused by sudden impact during flight)
- delamination of the propeller structure in the case of composite or wooden propellers,
- motor bearings,
- ESC abnormalities and loss of one of the electric phases,
- damage of the arm structure in the case of composite arms.

Figure 9 shows the idea of the piezo sensor and its mounting location. The piezo sensor will be mounted near the motor on the multicopter/VTOL arm. Two configurations were selected (Figure 9, No. 1 and 2)—mounting planes that were tested. This will allow the detection of a larger spectrum of failures.

## 6. The Experimental Studies

### 6.1. The Laboratory Testrig

**Figure 10:**
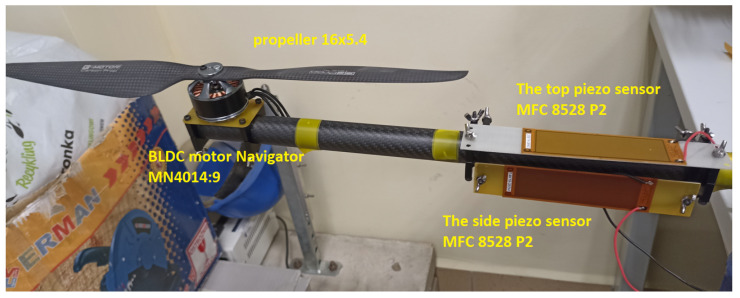
BLDC based drive unit with piezo-sensor.

**Figure 11:**
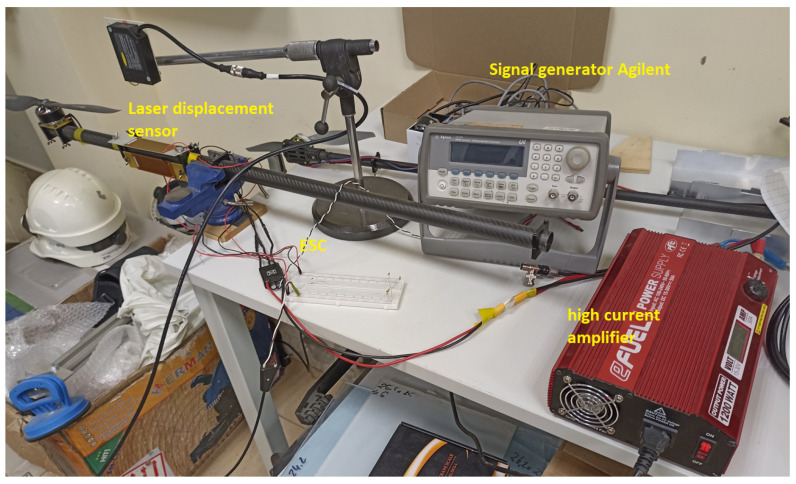
Laboratory test bench.

In this section, the performance of piezoelectric energy harvesting systems used to monitor of flight of a copter during the loss of lifting force by loss of one of the phases in the brushless motor is tested on a laboratory stand (see Figure 10 and Figure 11). To do this, the carbon fiber arm of the UAV containing the 3-phase brushless motor MN4014:9 developed by Navigator company and a propeller of the size of 16 × 5.4 has been chosen to further investigations.

Taking into account this fact, the arm of the UAV was filled with two macro fiber composites of type MFC 8528P2 which are located in two perpendicular planes versus the longitudinal axis of this arm. One of these piezos are located on the top of this arm while the second—one on side of this arm. In addition, to carry out experimental tests, the laboratory stand was retrofitted with a laser displacement sensor LQ10A65PUQ and the signal generator developed by Agilent company that are used to measure the vibration of this arm as well as to generate PWM signal directed to a BLDC motor, respectively.

### 6.2. Results

**Figure 12:**
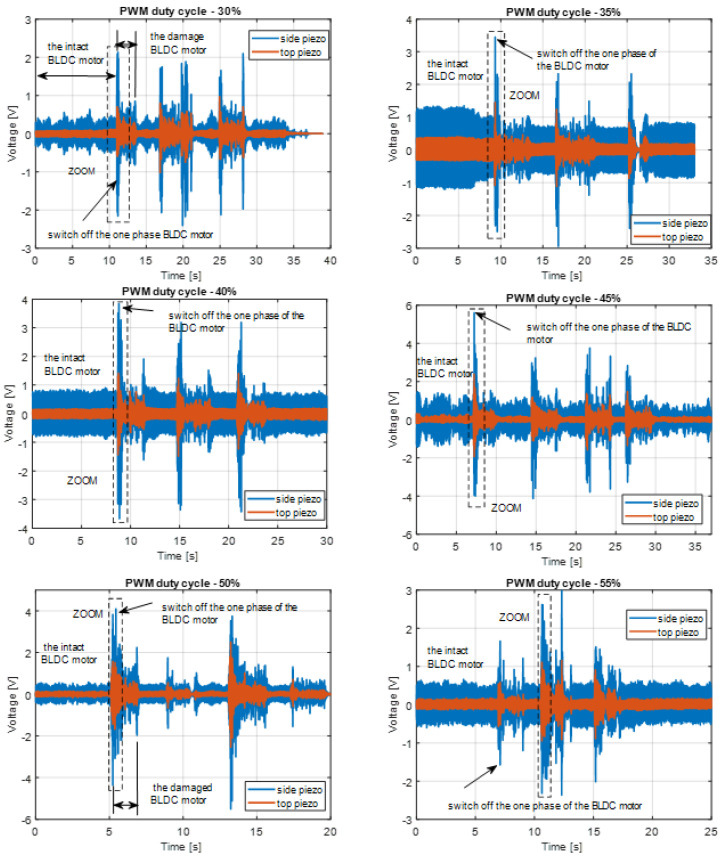
The comparison of voltage generated by the top piezo sensor and the side piezo sensor by various values of the PWM duty cycle in the range 30–55%.

In the first step, the experimental investigations were focused to assess the influence of the PWM signal duty cycle on the amplitude of voltage generated by both piezo-sensors as well as determining the proper location of these sensors on the structure. To do this, the chosen excitation signal in the limited range of duty cycle of 30–55% with step 5% is applied to the brushless motor in two stages. In the first step, this signal is applied to the intact motor (3 phases), while in the second one, to the damaged BLDC motor, which acts only with two phases. The measured and recorded voltage signals from both piezo sensors for both motors, intact and damaged, show that increasing the duty cycle of the PWM signal up to 45% leads to increasing amplitudes of these voltages (see Figure 12). Such behavior of the EH system is due to the fact that the vibration frequency of the system composed of the arm, motor, and propeller was more closer to the natural frequency of the arm where both piezo sensors are located. In the situation when the PWM duty cycle equals 50% and 55%, it can be observed that voltages generated by both EH systems are slightly decreasing, and it is due to the smaller bending stress of smart structures in the vicinity location of these piezo-sensors.

Further analysis of these diagrams shows that the energy harvesting system leads to faster detection of one-phase damage in the BLDC motor by using the side piezo sensor, as well as obtaining better performances in comparison to the top energy harvesting system containing the top piezo sensor. To confirm this conclusion, further investigations are focused on the analysis of the spectrum of voltage signals generated by both piezo-sensors for both the intact and damaged BLDC motor, respectively.

**Figure 13:**
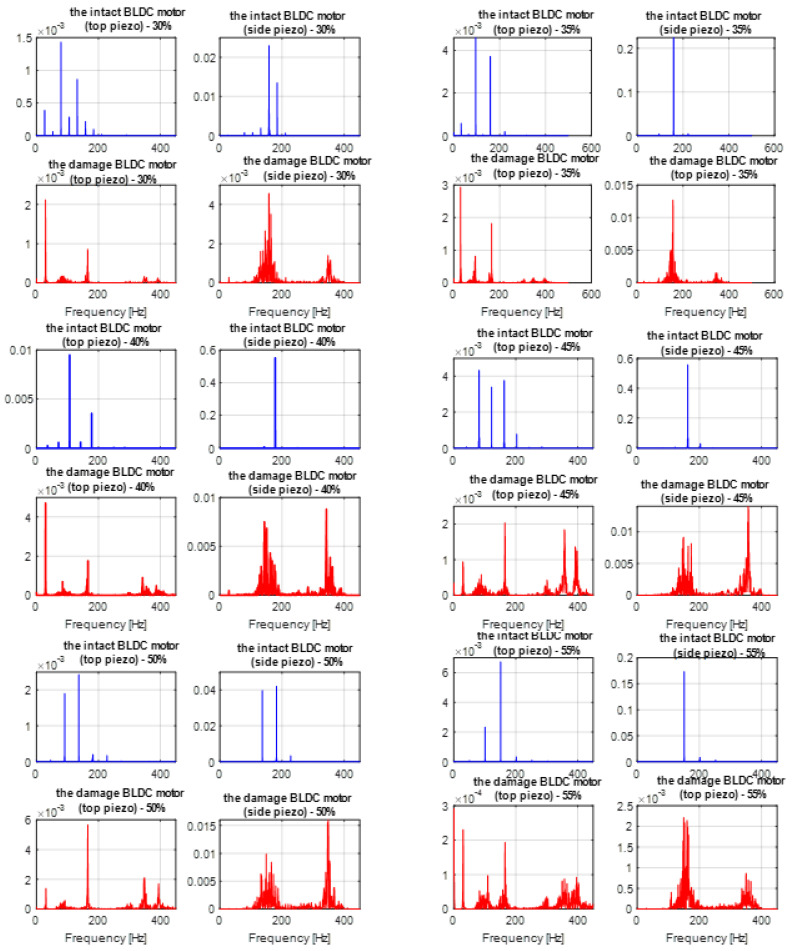
Frequency corresponding to voltage time series in Figure 12 for different values of the duty cycle of PWM signal applying to the BLDC motor.

Observing diagrams presented in Figure 13, especially for the intact motor and side piezo sensor, it can be shown that the recorded voltage signals are periodic, and higher frequencies of chosen arm vibrations lead to the increase in its stiffness and consequently to the increase in bending stress of this arm in the vicinity of its fixed end. The reason for this reaction is superharmonic vibrations, measured mainly by the side piezo, which additionally stimulate the energy harvesting system to generate higher voltages. A similar effect can be observed during the analysis of the spectrum of the randomly recorded voltages from the side piezo sensor where the damage to the motor occurred. Super harmonic frequency was close to 150 Hz. In addition, further analysis of these spectra plots allows the damage detection in the motor that it represents by voltage peak appearing on the diagram in frequency close to 350 Hz.

**Figure 14:**
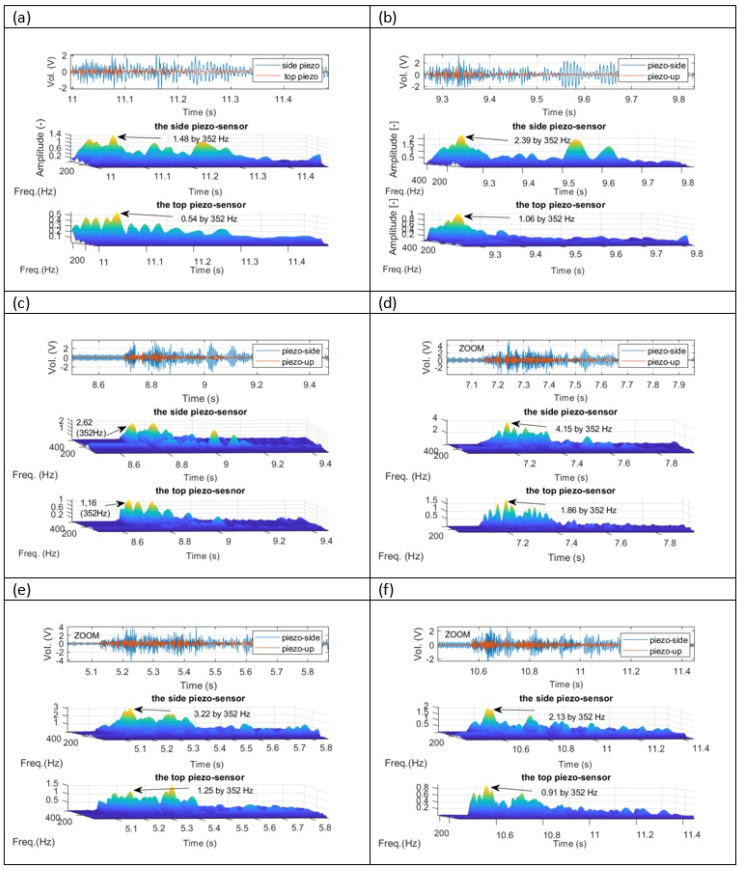
Wavelet analysis of the recorded voltages signals from both piezo composites sensors corresponding to response time in Figure 12 for damaged BLDC motor and various values of PWM duty cycle ((**a**) results for 30% duty cycle PWM signal, (**b**) results for 35% duty cycle PWM signal, (**c**) results for 40% duty cycle PWM signal, (**d**) results for 45% duty cycle PWM signal, (**e**) results for 50% duty cycle PWM signal, (**f**) results for 55% duty cycle PWM signal).

In the second step, further investigations are focused on the analysis of time-frequency diagrams that are generated for damage to brushless motors as well as for all PWM duty cycles in the range 30–55%. Taking into account Figure 13 and Figure 14 one more time, it can be observed that the side energy harvesting system at the moment of damage motor generated significantly higher amplitudes peak in frequency close to 352 Hz in comparison to the top energy harvesting system. In addition, it can be shown the highest amplitude was obtained for motor acting with PWM duty cycle equals 45%. As a result, it finally concluded that the proposed energy harvesting located on the side of the chosen arm of the UAV is sufficient for structural health monitoring of the BLDC motor during flight.

The presented results of experimental research showed a strong correlation between the moment of motor failure injection in a significant increase in the level of registered vibrations. During the laboratory tests, the influence of the location of the sensor (top or side) on the recorded vibrations was verified with the usage of a piezoelectric bar.

## 7. Conclusions

The paper presents an innovative method of detecting incorrect operation of the electric drive in a VTOL unmanned aerial vehicle in the quadwing configuration. A laboratory stand with the same drive as used for the VTOL propulsion system was developed and described. Based on that, a series of measurements were carried out for different piezoelectric positions. As shown in the waveforms of the measured vibration signals of the arm with the BLDC drive and damage simulation, it is possible to detect motor work anomalies on the basis of vibrations sensed by the piezo element. The voltage output signal from the piezo element can be used as a trigger—for example, on the analog input of the autopilot to change/start the appropriate UAV control algorithm—convenient for the emergency situation. The usage of a piezoelectric sensor and monitoring of vibration enables the control not only of sudden failures but also continuous monitoring of the technical condition of the propulsion system. The next step of the work will be testing other sizes of piezoelectrics with different characteristics and testing the operation of the sensor in the event of other types of drive-related failures (propeller delamination, propeller unbalance). It is also planned to perform flight tests and connect the drive failure detection system with the failure-tolerant control system. 

## Data Availability

Not applicable.

## References

[1] Gundlach G. (2011). Designing Unmanned Aircraft Systems: A Comprehensive Approach.

[2] Nonami K., Kendoul F., Suzuki S., Wang W., Nakazawa D. (2010). Autonomous Flying Robots, Unmanned Aerial Vehicles and Micro Aerial Vehicles.

[3] Valavanis K.P., Vachtsevanos G.J. (2015). Handbook of Unmanned Aerial Vehicles.

[4] Narazaki Y., Hoskere V., Chowdhary G., Spencer B.F. (2022). Vision-based navigation planning for autonomous post-earthquake inspection of reinforced concrete railway viaducts using unmanned aerial vehicles. Autom. Constr..

[5] Weng Y., Shan J., Lu Z., Lu X., Spencer B.F. (2021). Homography-based structural displacement measurement for large structures using unmanned aerial vehicles. Comput. Aided Civ. Infrastruct. Eng..

[6] Shan J., Liu Y., Cui X., Wu H., Loong C.N., Wei Z. (2022). Multi-level deformation behavior monitoring of flexural structures via vision-based continuous boundary tracking: Proof-of-concept study. Measurement.

[7] Yu R., Li P., Shan J., Zhu H. (2022). Structural state estimation of earthquake-damaged building structures by using UAV photogrammetry and point cloud segmentation. Measurement.

[8] Ambroziak L., Simha A., Pawluszewicz E., Kotta U., Bozko A., Kondratiuk M. Motor Failure Tolerant Control System With Self Diagnostics for Unmanned Multirotors. Proceedings of the 24th International Conference on Methods and Models in Automation and Robotics (MMAR).

[9] Caliskan F., Hajiyev C. (2016). Active Fault-Tolerant Control of UAV Dynamics against Sensor-Actuator Failures. J. Aerosp. Eng..

[10] Partridge M., Alwi H., Edwards C. Fault Tolerant Control Systems for Novel Tilt Wing UAV Platform. Proceedings of the 5th Conference on Control and Fault-Tolerant Systems (SYSTOL 2021).

[11] Suti A., Di Rito G., Galatolo R. (2022). Fault-Tolerant Control of a Dual-Stator PMSM for the Full-Electric Propulsion of a Lightweight Fixed-Wing UAV. Aerospace.

[12] Oldziej D., Gosiewski Z. (2013). Modelling of Dynamic and Control of Six-Rotor Autonomous Unmanned Aerial Vehicle, Solid State Phenomena.

[13] Setlak L., Kowalik R. (2018). Studies of 4-rotor unmanned aerial vehicle UAV in the field of control system. Proceedings of the 22nd International Conference on Circuits, Systems, Communications and Computers (CSCC 2018).

[14] Ferrao I.G., da Silva S.A., Pigatto D.F., Branco K.R.L.J.C. GPS Spoofing: Detecting GPS fraud in unmanned aerial vehicles. Proceedings of the 2020 XVIII Latin American Robotics Symposium (LARS-SBR-WRE 2020).

[15] Mungula R. (2020). A GPS-aided inertial navigation system in direct configuration. J. Appl. Res. Technol..

[16] Shvets V., Ilnytska S., Kutsenko O. (2019). Application of Computer Modelling in Adaptive Compensation of Interferences on Global Navigation Satellite Systems.

[17] Cheng P., Gao Z., Qian M., Lin J. Active fault tolerant control design for UAV using nonsingular fast terminal sliding mode approach. Proceedings of the 2018 Chinese Control And Decision Conference (CCDC).

[18] Zhao X.F., Li X.W., Zhang J.Z., Zhang Z. (2022). Active Fault-Tolerant Control Scheme for Unmanned Air-Ground Attitude System with Time-Varying Delay Faults. Appl. Sci..

[19] Zhou Y.M., Zhao H.R., Liu Y.L. (2020). An evaluative review of the VTOL technologies for unmanned and manned aerial vehicles. Comput. Commun..

[20] Poorina B.S., Javid A. (2020). IOT Monitored Brushless DC Motor Speed Control Using Arduino. Int. J. Adv. Eng. Res. Sci..

[21] Wu Y., Jiang B., Wang Y. (2020). Incipient winding fault detection and diagnosis for squirrel-cage induction motors equipped on CRH trains. ISA Trans..

[22] Ambroziak L., Ciężkowski M., Wolniakowski A., Romaniuk S., Bożko A., Ołdziej D., Kownacki C. (2022). Experimental tests of hybrid VTOL unmanned aerial vehicle designed for surveillance missions and operations in maritime conditions from ship-based helipads. J. Field Robot..

[23] Ambroziak L., Kownacki C., Bożko A. Hybrid VTOL UAV Autonomous Operations from Mobile Landing Pad. Proceedings of the 26th International Conference on Methods and Models in Automation and Robotics: MMAR 2022.

[24] Koszewnik A., Oldziej D., Amaro M.B. (2022). Parameter Optimization of a Magnetic Coupled Piezoelectric Energy Harvester with the Homogenized Material—Numerical Approach and Experimental Study. Sensors.

[25] Litak G., Margielewicz J., Gaska D., Wolszczak P., Zhou S. (2021). Multiple Solutions of the Tristable Energy Harvester. Energies.

[26] Koszewnik A., Lesniewski K., Pakrashi V. (2021). Numerical Analysis and Experimental Verification of Damage Identification Metrics for Smart Beam with MFC Elements to Support Structural Health Monitoring. Sensors.

[27] Koszewnik A., Oldziej D. (2019). Performance assessment of an energy harvesting system located on a copter. Eur. Phys. J. Spec. Top..

[28] Koszewnik A., Gosiewski Z. (2016). Quasi-optimal locations of piezo-elements on a rectangular plate. Eur. Phys. J. Plus.

